# PAK4 confers the malignance of cervical cancers and contributes to the cisplatin-resistance in cervical cancer cells via PI3K/AKT pathway

**DOI:** 10.1186/s13000-015-0404-z

**Published:** 2015-09-28

**Authors:** Xiang-Rong Shu, Jing Wu, He Sun, Li-Qun Chi, Jin-Huan Wang

**Affiliations:** School of Pharmaceutical Science and Technology, Tianjin University, No. 92, Weijin Road, Nankai District, Tianjin, 300072 China; Tianjin Huanhu Hospital, No.122, Qixiangtai Road, Hexi District, Tianjin, 300060 China; Haidian Maternal & Child Health Hospital, Beijing, 10080 China

## Abstract

**Background:**

Multiple protein or microRNA markers have been recognized to contribute to the progression and recurrence of cervical cancers. Particular those, which are associated with the chemo- or radio-resistance of cervical cancers, have been proposed to be promising and to facilitate the definition for cervical cancer treatment options.

**Methods:**

This study was designed to explore the potential prognosis value of p21-activated kinase (PAK)-4 in cervical cancer, via the Kaplan–Meier analysis, log-rank test and Cox regression analysis, and then to investigate the regulatory role of PAK4 in the cisplatin resistance in cervical cancer cells, via the strategies of both PAK4 overexpression and PAK4 knockout.

**Results:**

It was demonstrated that PAK4 was upregulated in cervical cancer tissues, in an association with the cancer’s malignance variables such as FIGO stage, lymph node or distant metastasis and the poor histological grade. The high PAK4 expression was also independently associated with poor prognosis to cervical cancer patients. Moreover, PAK4 confers cisplatin resistance in cervical cancer Hela or Caski cells. In addition, the PI3K/Akt pathway has been implicated in the PAK4-confered cisplatin resistance. And the PI3K/Akt inhibitor, LY294002, markedly deteriorated the cisplatin-mediated viability reduction of Hela or Caski cells, indicating the involvement of PI3K/Akt pathway in the cisplatin resistance in cervical cancer cells.

**Conclusion:**

This study has confirmed the significant prognostic role of PAK4 level in cervical cancer patients and has recognized the regulatory role in cervical cancer progression. Moreover, our study has indicated that PAK4 also confers the chemoresistance of cervical cancer cells in a PI3K/Akt-dependent way. Thus, our study indicates PAK4 as a promising marker for cervical cancer treatment.

## Background

Cervical cancer records the third most common women malignancy, with an estimated global incidence of over 500,000 new cases [1], and leads secondly the death cause of women world widely, with an estimated 530,000 deaths per year [2]. Multistep processes and molecular markers have been confirmed to be involved in the tumorigenesis, invasiveness of cervical cancers [3]. Although radiotherapy, chemotherapy and surgery have recently been standardized for patients with cervical cancer, clinical outcomes still vary significantly. Therefore, it is important to expand the knowledge of the molecular pathways and markers of cervical cancers to identify prognostic markers and to improve therapeutic strategies. Cervical cancer is clinically staged according to such prognostic factors as clinical stage at diagnosis time, tumor size, vascular invasion, and adjacent/lymphatic metastasis. And such staging define the treatment option for single surgery or for multidisciplinary treatments with either concurrent chemoradiation or with neoadjuvant chemotherapy followed by surgery [4].

The etiology of cervical cancer has been largely attributed to infection of human papillomavirus (HPV) [5, 6]. However, HPV infection does not necessarily lead to such cancer [7]. And accumulating studies gaining insight into other molecular characterization of it have identified many novel biological factors, which directly or indirectly regulate cell cycle, apoptosis, angiogenesis, or invasive or metastatic potential of cervical cancers [8–10]. Moreover, since the therapeutic resistance is a common phenomenon in cervical cancers, particularly in patients with advanced, recurrent, and metastatic disease [7]. Thus, biomarkers of proteins [11, 12] and microRNAs [13–15], which are associated with the chemo- radio-resistance of cervical cancer have been proposed to be promising and to facilitate the definition for cervical cancer treatment options.

The small GTPases, i.e. Ras, Rho, Rac and Cdc42 are a family of G-proteins in the cytosol function independently as a hydrolase enzyme (bind and hydrolyze guanosine triphosphate (GTP)). p21-activated kinases (PAKs) are a family of serine/threonine protein kinases (PAK1-6) which are best characterized downstream effectors of Rac and Cdc42 [16]. PAKs have increasingly recognized to be overexpressed and/or hyperactivated in several human tumors such as breast cancer, colon cancer, lung cancer and gastric cancer [17, 18], closely correlating with cancer development. PAKs are significantly relevant to tumorigenesis by regulating the Ras-induced cell cycle progression and metabolism [19, 20], epithelial–mesenchymal transition [21] and angiogenesis [22]. Besides, PAK4 has been recognized to modulate the cancer migration and invasion via interacting with Met [23] or with DGCR6L [18]. Moreover, PAK4 has recently been found to confer cisplatin resistance in gastric cancer cells [24] or in glioma [25]. However, the oncogenic role of PAK4 in cervical cancer has not been reported.

This study was designed to explore the potential prognosis value of PAK4 in cervical cancer, and then to investigate the regulatory role of PAK4 in the cisplatin resistance in cervical cancer cells. Our results demonstrate that PAK4 is closely associated with the development and progression of cervical cancer and confers cisplatin resistance in cervical cancer cells.

## Methods

### Cervical cancer patients

93 patients with cervical cancer of stage IB–IIIA, who were registered between April 2013 and November 2014 in the Department of Gynecology, Tianjin Huanhu Hospital were included in the present study. Among them, 68 cases were squamous cell carcinoma, the other 25 cases were adenocarcinoma, and 67 cases were HPV-positive, whereas the other 26 cases were HPV-negative. Detailed clinic-pathological information was shown in table 1. Fresh cervical cancer tissues and their matched peri-tumor tissues (2 cm away from the boundary of cervical cancer tissues) were collected from the 93 cervical cancer patients underwent surgery and were immediately frozen at −80 °C before use. Each patient was pathologically confirmed by two pathologists, had no preoperative chemotherapy or radiotherapy, and was staged according to the criteria of the International Federation of Gynecology and Obstetrics (FIGO). External beam radiotherapy was administered, with large-field radiation dose of 40–45 Gy to the whole pelvis, with 1.8-2 Gy/fraction and five fractions weekly. For patients with Stage IIb or greater disease, a small-field parametrial boost was given to a dose of up to 60–90 Gy using a parallel-opposed anteroposterior field. For the chemotherapy, cisplatin was given intravenously with 40 mg/m^2^ once a week during the external beam radiotherapy for the 44 patients with a FIGO stage of Ib or IIa. For the 49 patients with a FIGO stage of IIb or IIIa, two cycles of 60 mg/m^2^ docetaxel and 80 mg/m^2^ cisplatin during the external beam radiotherapy. Overall survival (OS) time was calculated from the date of the initial surgical operation to death. Follow-up for all patients was performed every 2 months for the first 2 years, every 4 months for the third year, and every 6 months for the 4 to 5 years. This study was approved by the Research Ethics Committee of the Tianjin Huanhu Hospital. Written informed consent was obtained from each patient in this study.

**Table 1:** Association PAK4 expression with clinicopathological features of human cervical cancer

Characteristics	No. (*n* = 95)	High PAK4		Low PAK4		*P* value
		N	%	N	%	
Age (years)	93					0.42131
<65	56	24	55.81	32	64.00	
≥65	37	19	44.19	18	36.00	
Tumor size (cm)						0.59763
<4	46	20	46.51	26	52.00	
≥4	47	23	53.49	24	48.00	
HPV						0.99205
Positive	67	31	72.09	36	72.00	
Negative	26	12	27.91	14	28.00	
Tumor histology						0.25219
Squamous	68	29	67.44	39	78.00	
Adenocarcinoma	25	14	32.56	11	22.00	
FIGO stage						0.02888*
Ib	24	6	13.95	18	36.00	
IIa	20	8	18.60	12	24.00	
IIb	25	13	30.23	12	24.00	
IIIa	24	16	37.21	8	16.00	
LN metastasis						**0.00525**
Positive	38	24	55.81	14	27.45	
Negative	56	19	44.19	37	72.55	
Distant metastasis						**0.02105**
Positive	26	17	39.53	9	18.00	
Negative	67	26	60.47	41	82.00	
Histological grade						**0.03464**
Poor	41	24	55.81	17	34.00	
Well/moderade	52	19	44.19	33	66.00	

*: with significance

*: with significance. 

### Cell culture and treatment

Human cervical cancer Hela and Caski cells were purchased from American Type Culture Collection (ATCC) (Rockville, MD, USA) and were cultured in Dulbecco’s Modified Eagle Medium (DMEM) (for Hela cells) or RPMI-1640 medium (for Caski cells), which was supplemented with 10 % fetal bovine serum (FBS) (GIBCO, Rockville, MD, USA), with 100 U/mL penicillin and 100 μg/mL streptomycin (CSPC, Shijiazhuang, China). Cells were incubated in a humidified atmosphere at 37 °C in 5 % CO_2_. For the cisplatin (Sigma-Aldrich, St. Louis, MO, USA) treatment, 85 % or higher confluent Hela or Caski cells were updated with DMEM or RPMI-1640 medium supplemented with 2 % FBS, and with 5 μM (for Hela cells) or 10 μM (for CaSki cells) cisplatin for 12, 24 or 48 h; For the PAK4 overexpression, the open reading frame (ORF) of PAK4 (NM_005884.3) was amplified by PCR with Phusion polymerase (New England Biolabs, Ipswich, MA, USA) and with the primers (Forward primer: 5′-ATG TTT GGG AAG AGG AAG AAG C-3′ and Reverse primer: 5′-TCA TCT GGT GCG GTT CTG GCG-3′). The ORF sequence was then cloned into the pcDNA3.1(+) vector (Invitrogen, Carlsbad, CA, USA), with Hind III (New England BioLabs, Beverly, MA, USA) and BamH I (New England BioLabs, Beverly, MA, USA) as restriction enzymes, and with the chloramphenicol acetyltransferase (CAT) as a control for PAK4. Hela or Caski cells with more than 85 % confluence were transfected with the recombinant pcDNA3.1(+)-PAK4 or pcDNA3.1(+)-Con plasmid with Lipofectamine 2000 (Invitrogen, Carlsbad, CA, USA). For the PAK4 knockout, the PAK4-specific siRNA (siRNA-PAK4) or siRNA-Con (Santa Cruz Biotechnology, Santa Cruz, CA, USA) with 25 or 50 nM were transfected with Lipofectamine RNAiMax (Invitrogen, Carlsbad, CA, USA) into the Hela or Caski cells to abrogate the HIWI expression. Phosphoinositide 3-kinase/ RAC-alpha serine/threonine-protein kinase (PI3K/Akt) inhibitor, LY294002 (Thermo Scientific, Rockford, IL, USA) was utilized with 10 or 20 nM (Hela cells) or with 15 or 30 nM (Caski cells) to block the PI3K/Akt pathway.

### Quantitative analysis of PAK4 with RT-qPCR

To investigate the expression of PAK4 on mRNA level, Real-time quantitative polymerase chain reaction (RT-qPCR) was performed with PAK4-specific primers (Forward primer: 5′-CAG GGA AGG CAG GCA GCC GA-3′ and Reverse primer: 5′-CCT GTC ACC ACT GCC GCC AC-3′) with the mRNA samples from cervical cancer tissue specimens and cervical cancer Hela or Caski cells. mRNA samples were prepared from the cervical cancer tissues, peritumor tissues, or from Hela or Caski cells with the TRIzol reagent (Invitrogen, Carlsbad, CA, USA) according to the product’s manual, were supplemented with Rnase inhibitor (Takara, Tokyo, Japan), and were stored at −80 °C before use. RT-qPCR procedure was performed with SYBR green OneStep RT-PCR Kit (Takara, Tokyo, Japan) according to the manufacturer’s manual. PAK mRNA level was calculated and was presented as the relative level of PAK4 to β-actin (as control) by ∆∆ Ct method [26].

### Cell viability assay with MTT

Cell viability was examined with methyl thiazolyl tetrazoliym assay (MTT). Briefly, Hela or Caski cells which were plated in 96-well plates with 85-90 % confluence post the treatment with cisplatin, post the plasmid or siRNA transfection or (and) post the treatment with LY294002 were updated with 50 μl MTT solution for a incubation at 37 °C for 2 h. Then cells were updated with 150 μl DMSO was added to dissolve the precipitate completely at room temperature. The optical density was measured at 570 nm using a spectrophotometer (Bio-Rad, Hercules, CA, USA).

### Western blot analysis

Hela or Caski cells, post treatment, were harvested with a cell scraper and were homogenized in an ice-cold Cell lysis buffer (Bio-Rad, Hercules, CA, USA). Cellular lysates were centrifugated with 12,000 × g for 30 min at 4 °C, and the supernatant was collected. Protein samples were separated with 10 % (w/v) SDS-PAGE gel and were transferred to a nitrocellulose membrane (Millipore, Bedford, MA, USA). For analysis of PAK4, AKT with or without phosphorylated Ser473, blots were incubated with rabbit polyclone antibody against human PAK4 (diluted with 5 % BSA to 1: 500, PA5-15120, Thermo Scientific, Rockford, IL, USA), against PAK4 with phosphorylated Ser474 (diluted with 5 % BSA to 1: 500, PA5-17636, Thermo Scientific, Rockford, IL, USA), against AKT with (diluted with 5 % BSA to 1: 500, No. 4685, Cell Signaling Technology Inc., Danvers, MA, USA) or without phosphorylated Ser473 (diluted with 5 % BSA to 1: 500, No. 4060, Cell Signaling Technology Inc., Danvers, MA, USA) or against β-actin (diluted with 5 % BSA to 1: 1000, No.1320, Sinobio, Beijing, China) respectively. Then, the specific binding on the membrane was probed with Horseradish Peroxidase (HRP)-labeled anti-rabbit secondary antibody (diluted with 5 % BSA to 1: 1000, #7071, Cell Signaling Technology Inc., Danvers, MA, USA) and with enhanced chemiluminescence (ECL) detection kit (Amersham Pharmacia Biotech, Amersham, UK).

### Statistical analysis

Statistical analysis was conducted using the GraphPad Prism (GraphPad Software, La Jolla, CA, USA). The chisquare test was used to evaluate the association of PAK4 overexpression with clinic-pathological characteristics. Kaplan–Meier analysis and log-rank test were utilized to curve the overall survical of cervical cancer patients. The univariate Cox regression was performed to examine the influence of each clinic-pathological characteristic on patient survival. And the multivariate analysis with Cox proportional hazards model for each variable that were significant in the univariate analysis. *P* < 0.05 or less was considered as statistically significant.

## Results

### Upregulated PAK4 in cervical cancer, correlating with the cancer’s malignance

**Fig. 1:**
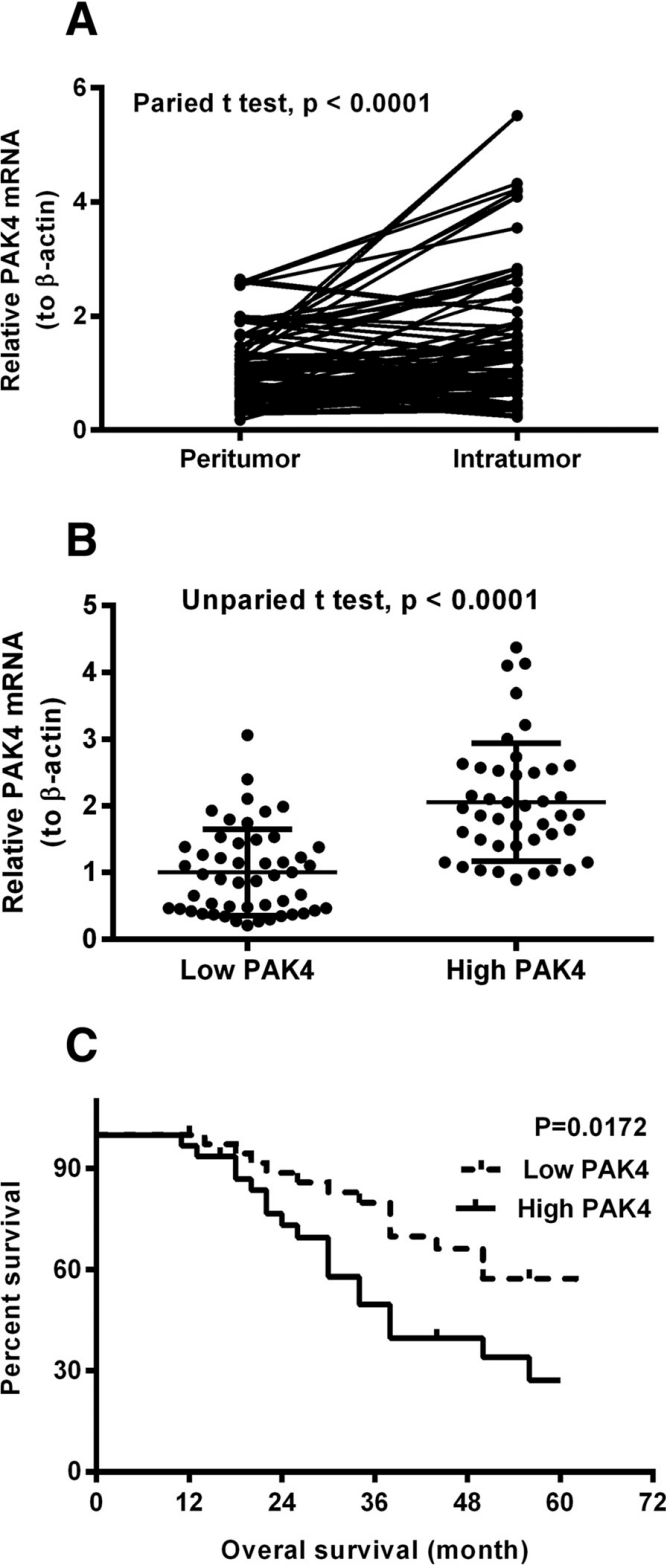
PAK4 is upregulated in cervical cancer specimens and associates with poor overall survival. **a** Upregulated PAK4 mRNA level in the intra-tumor specimens than in the peritumor specimens of cervical cancer patients, with paired *t* test; **b** Increased PAK4 mRNA level in cervical cancer specimens, with unpaired *t* test; **c** Kaplan-Meier survival analysis for high PAK4 expression level (>1) versus low PAK4 expression level (≤1) in patients with cervical cancer. Statistical significance was considered when *p* < 0.05

There were total of 93 cases of cervical cancer patients were involved in this study. As shown in table 1, these patients were aged from 30 to 75 year, with a median age of 53 years. The detailed tumor clinic-pathological characteristics of all patients, such as tumor size, HPV infection, tumor histology and histological grade, FIGO stage, distant and lymph node metastasis are summarized in table 1. And the PAK4 expression in mRNA level in the peritumor tissue (more than 2 cm from tumor tissue) and in tumor tissue in each patient was examined with RT-qPCR method. Figure 1 demonstrated that the relative PAK4 mRNA level (with peritumor mRNA sample as control) was significantly higher in the tumor specimens, via the two-tailed paired *t* test (*p* < 0.0001, R2 = 1802, 95 % confidence interval (0.2545 to 0.6573)). We then re-grouped those samples according to their PAK4 levels as high PAK4 group (*n* = 43) and low PAK4 group (*n* = 50) (the patient with the relative PAK4 mRNA level higher than “1” in the tumor sample than in the peritumor sample was classified into the high PAK4 group), and the unpaired *t* test indicated a significant difference between the two group (*p* < 0.0001, Fig. 1b).

To recognize the association between the PAK4 high expression and each clinic-pathological characteristic, we analyzed the statistical difference in age, tumor size, HPV infection, tumor histology, FIGO stage, lymph node metastasis, distant metastasis and histological grade between the high PAK4 and low groups with chi-square test. And it was indicated in table 1 that there was no significant difference in age, tumor size, HPV infection, or tumor histology between the two groups of patients. However, the high PAK4 expression was markedly associated with the FIGO stage, lymph node metastasis, distant metastasis and histological grade of these cervical cancer patients. Those patients with higher FIGO stage (*p* = 0.00822), with lymph node (*p* = 0.00525) or distant (*p* = 0.02105) metastasis, or with poor histological grade (*p* = 0.03464) presented significantly higher level of PAK4 mRNA in their cervical cancer specimens.

### High PAK4 expression associates with poor prognosis to cervical cancer patients

To recognize the prognostic value of PAK4 expression in patients with cervical cancer, we then investigated the association between PAK4 expression and overall survival of cervical cancer patients by Kaplan-Meier analysis and log-rank test. During the follow-up period, 37 of the 93 patients (39.78 %) died within 60 months. As shown in Fig. 1c, the overall survival time was significantly less for patients with higher PAK4 level than those with lower PAK4 expression (log-rank test: *P* = 0.0172). And the univariate analysis demonstrated that the FIGO stage (*P* = 0.0026), the lymph node metastasis (*P* = 0.0048) and the histological grade (*P* = 0.0375), and PAK4 expression (*P* = 0.0014) were significantly associated with overall survival of cervical cancer patients (Table 2). However, there was no significant association was found between each of other clinic-pathological characteristics and the overall patient survival. In addition, the multivariate analysis for each significant variable in the univariate analysis deomonstrated that the FIGO stage (*P* = 0.0097), the lymph node metastasis (*P* = 0.0124), or the PAK4 expression (*P* = 0.0073) was independently associated with the poor prognostic overall survival for patients with cervical cancer (Table 3).

**Table 2:** Univariate analysis of prognostic factors in cervical cancer patients

Variables	Univariable analysis		
	Hazard ratio	95 % Confidence Interval	*P* value
Age			
< 65 vs ≥ 65	1.261	(0.714-1.906)	0.4362
Tumor size (cm)			
< 4 vs ≥4	1.179	(0.706-1.942)	0.4523
HPV			
Positive vs Negative	0.875	(0.437-1.691)	0.7831
Tumor histology			
Squamous vs Others	0.696	(0.354-1.342)	0.2874
FIGO stage			
Ib-IIa vs IIb-IIIa	**2.226**	**(1.416-3.752)**	0.0026*
LN metastasis			
Positive vs Negative	**2.042**	**(1.318-3.265)**	**0.0048**
Distant metastasis			
Positive vs Negative	1.660	(0.980-2.945)	0.0522
Histological grade			
Poor vs Well/moderate	**1.762**	**(1.130-2.706)**	**0.0375**
PAK4 expression			
High vs Low	**2.217**	**(1.435-3.562)**	**0.0014**

*: with significance

**Table 3:** Multivariate analysis of prognostic factors in cervical cancer patients

Variables	Multivariable analysis		
	Hazard ratio	95 % Confidence Interval	*P* value
FIGO stage			
Ib-IIa vs IIb-IIIa	**2.53**	**(1.16-4.68)**	**0.0097**
LN metastasis			
Positive vs Negative	**1.81**	**(1.22-2.83)**	**0.0124**
Histological grade			
Poor vs Well/moderate	**1.45**	**(0.95-2.40)**	**0.0683**
PAK4 expression			
High vs Low	**3.21**	**(1.47-5.35)**	**0.0073**

### PAK4 confers cisplatin resistance in cervical cancer cells *in vitro*

**Fig. 2:**
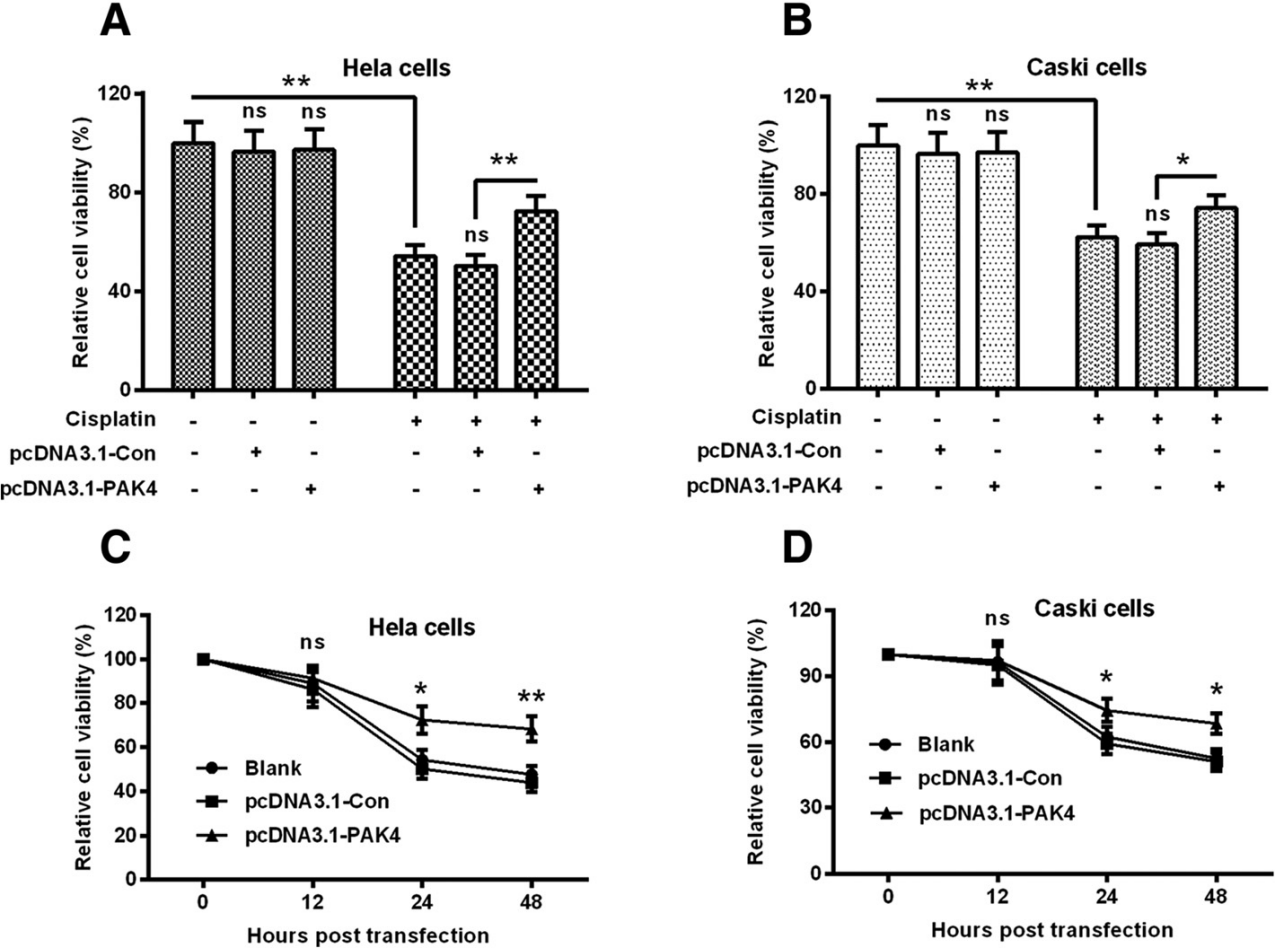
Overexpressed PAK4 ameliorates the cisplatin-induced viability reduction of cervical cancer cells*.* **a** and **b**: The percent viability of Hela (**a**) and CaSki (**b**) cells which were transfected with control pcDNA3.1 (pcDNA3.1-Con) or pcDNA3.1-PAK4, with or without the treatment with 5 μM (for Hela cells) or 10 μM (for CaSki cells) cisplatin for 24 h; **c** and **d**: Time-dependence of the influence of the transfection with pcDNA3.1-Con or with pcDNA3.1-PAK4 on the viability of Hela (**c**) and CaSki (**d**) cells, in the presence of 5 μM (for Hela cells) or 10 μM (for CaSki cells) cisplatin. All results were averaged for triple independent experiments. *P < 0.05, **P < 0.01 or ns: no significance

To further investigate the association of PAK4 with the poor prognosis of cervical cancer patients, we examined the influence of PAK4 overexpression on the sensitivity of Hela and Caski cells to cisplatin. It was indicated in Fig. 2a that the PAK4 overexpression posed no obvious influence on the viability of Hela cells, compared to the control plasmid and the blank control. However, the cellular viability decreased dramatically post the 5 μM cisplatin treatment (Column 4 vs Column 1, *p* < 0.01). Moreover, the transfection with pcDNA3.1-PAK4 markedly ameliorated the viability reduction by the cisplatin treatment, compared to the transfection with the pcDNA3.1-Con plasmid (Column 6 vs Column 5, p < 0.01), whereas there was no significant difference between the pcDNA3.1-Con-transfected and blank Hela cells (Column 5 vs Column 4). Then we re-examined such influence of PAK4 overexpression in Caski cells, and results demonstrated that the viability of the pcDNA3.1-PAK4-transfected Caski cells was also significantly higher than the pcDNA3.1-Con-transfected or the blank Caski cells (*p* < 0.05, Column 6 vs Column 5, Fig. 2b). In addition, we examined the time-dependence of the cellular viability amelioration by PAK4 overexpression in the two types of cells. It was indicated that such amelioration was significant at 24 or 48 h post the transfection in both Hela (*p* < 0.05 or *p* < 0.01, Fig. 2c) and Caski (either *p* < 0.01, Fig. 2d) cells. Therefore, PAK4 inhibited the sensitivity of Hela and Caski cells to cisplatin.

**Fig. 3:**
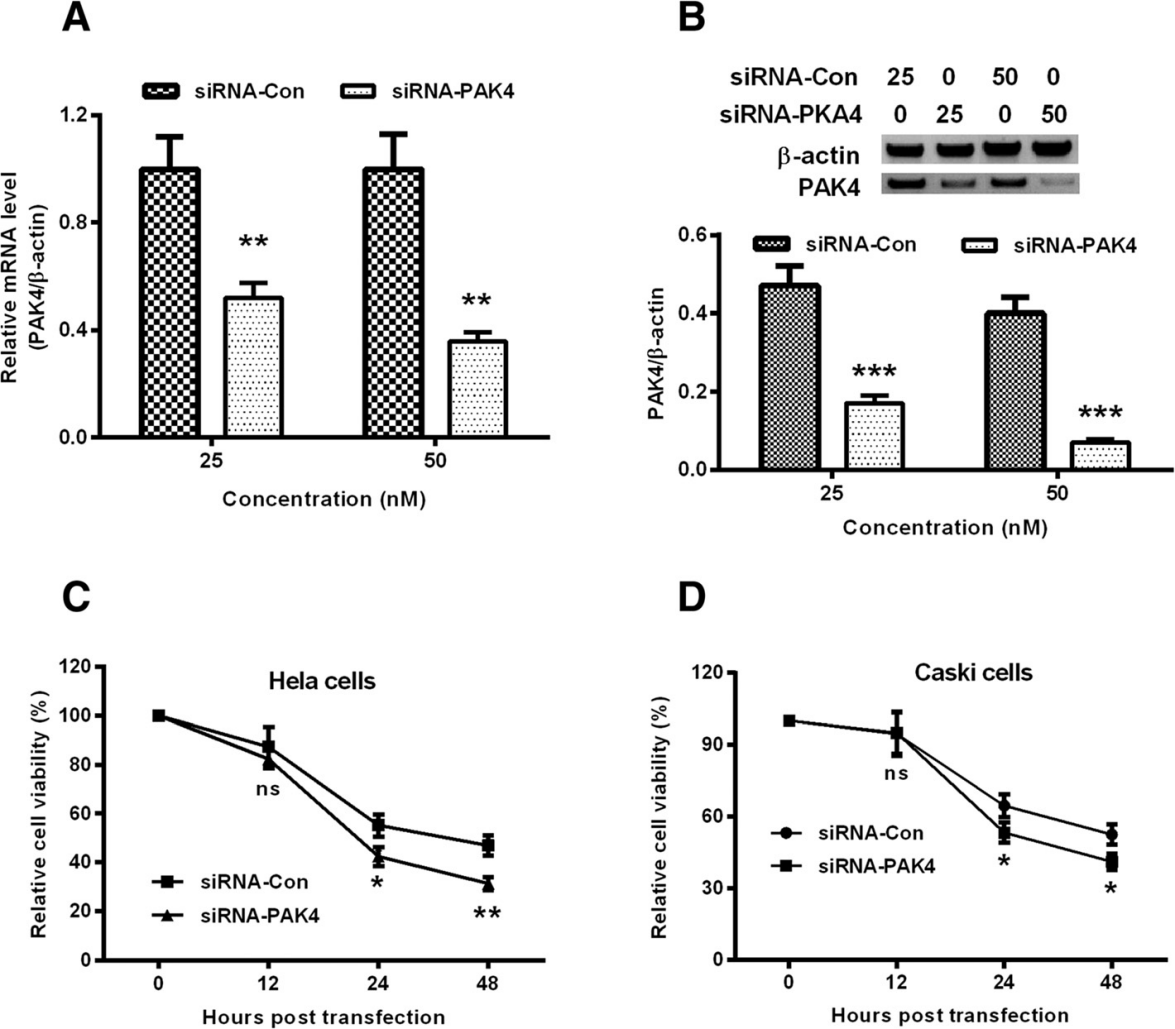
PAK4 knockdown inhibits the cisplatin resistance in cervical cancer cells. **a**: PAK4 mRNA level in Hela cells which were transfected with the PAK4-targeted siRNA (siRNA-PAK4) or with the control siRNA (siRNA-Con) with a concentration of 25 or 50 nM; **b**: Western blot analysis of PAK4 in protein level in the siRNA-PAK4- or siRNA-Con-transfected Hela cells; C and D: Influence of the transfection with siRNA-PAK4 or with siRNA-Con on the viability of Hela (**c**) and CaSki (**d**) cells, post the treatment with5 μM (for Hela cells) or 10 μM (for CaSki cells) cisplatin for 12, 24 or 48 h. Each value was averaged for triple independent results. *P < 0.05, **P < 0.01 or ***P < 0.001, ns: no significance

To further confirm the effect of PAK4 on cisplatin efficacy, we knockdown the PAK4 expression, and re-evaluated the cisplatin-mediated viability reduction in both Hela and Caski cells. Results indicated that PAK4 was significantly downregulated in both mRNA (*p* < 0.01 for 25 or 50 nM, Fig. 3a) and protein levels (*p* < 0.001 for 25 or 50 nM, Fig. 3b). And then we also re-examined the cisplatin-mediated viability reduction in both types of cells. It was demonstrated in Fig. 3c that the transfection with 50 nM siRNA-PAK4 significantly aggravated the cisplatin-mediated viability reduction at either 24 or 48 h post treatment (*p* < 0.05 or *p* < 0.01). And Fig. 3d also indicated that the siRNA-PAK4 transfection markedly deteriorated the cisplatin-mediated viability reduction at either 24 or 48 h post treatment (*p* < 0.05 respectively). Therefore, the PAK4 knockdown sensitized Hela and Caski cells to cisplatin.

### PI3K/Akt-dependent pathway is implicated in the PAK4-confered cisplatin resistance

**Fig. 4:**
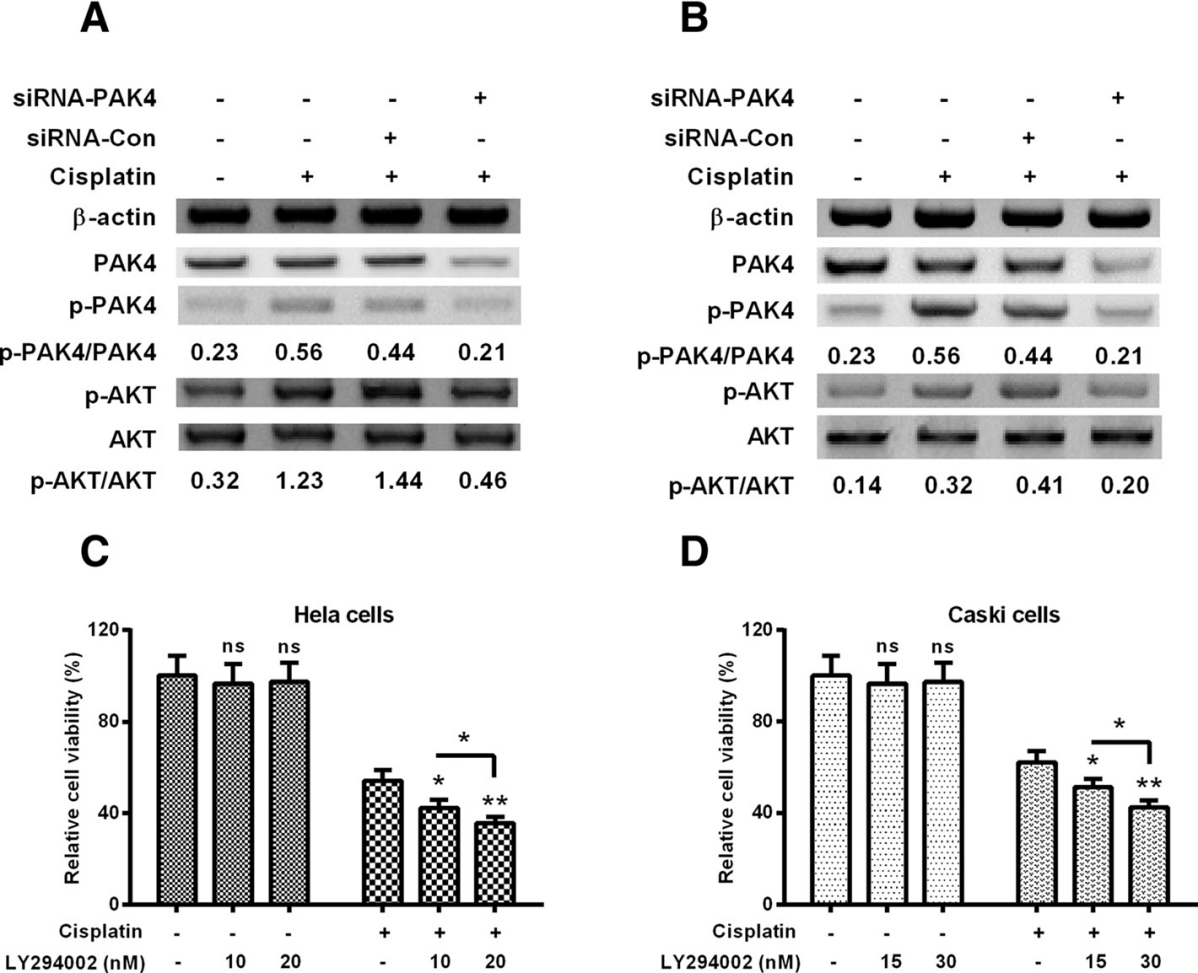
PI3K/Akt-dependence of the PAK4-mediated cisplatin resistance in cervical cancer cells. **a** and **b**: Phosphorylation of PI3K/Akt in Hela (**a**) and CaSki (**b**) cells, which were treated with 5 (for Hela cells) or 10 μM (for CaSki cells) cisplatin and with 10 or 20 nM (for Hela cells), or with 15 or 30 nM (for Caski cells), LY294002; **c** and **d**: Influence of the treatment with 10 or 20 nM LY294002 in Hela cells, or the treatment with 15 or 30 nM LY294002 in Caski cells on the cisplatin-mediated (5 μM for Hela cells or 10 μM for CaSki cells) viability reduction for 12, 24 or 48 h. Experiments were repeated in triplicate independently. *P < 0.05, **P < 0.01 or ns: no significance

To explore the mechanism underlying PAK4-induced cisplatin resistance in cervical cancer cells, we examined the activation of PI3K/Akt pathway in the cisplatin-treated Hela and Caski cells, with or without the transfection with siRNA-PAK4 or siRNA-Con. The western blotting results demonstrated that the PAK4 level was not markedly regulated by the 5 μM cisplatin treatment in both cell lines, whereas the phospholylated PAK4 was promoted by the 5 μM cisplatin treatment (Column 1 and 2, Fig. 4a and b). However, such both PAK4 and p-PAK4 was significantly downregulated by the transfection with siRNA-PAK4 (Column 3 and 4, Fig. 4a and b). Moreover, the phosphorylated AKT was significantly promoted by the cisplatin treatment in Hela cells. However, the promotion to the phosphorylated AKT was also inhibited by the transfection with 50 nM siRNA-PAK4, compared with the transfection with 50 nM siRNA-Con (Fig. 4a). And such promotion to the phosphorylated AKT and the inhibition by siRNA-PAK4 were also confirmed in Caski cells (Fig. 4b). To investigate the role of AKT phosphorylation in the cisplatin resistance in cervical cancer cells, we then measured the viability of cisplatin-treated Hela and Caski cells, in the presence of PI3K/Akt inhibitor, LY294002. Figure 4c demonstrated that there was no significant regulation on the viability of Hela cells by the treatment with 10 or 20 nM LY294002. However, the LY294002 treatment markedly deteriorated the cisplatin-mediated viability reduction of Hela cells with a concentration of 10 (*p* < 0.05) or 20 nM (*p* < 0.01), dose-dependently (*p* < 0.05). And such effect was repeated in Caski cells, either concentration of 15 or 30 nM LY294002 markedly aggravated the cellular viability reduction (*p* < 0.05 or *p* < 0.01, Fig. 4d), dose-dependently (*p* < 0.05). Thus, our results confirmed the involvement of PI3K/Akt-dependent pathway in the PAK4-confered cisplatin resistance.

## Discussion

PAK4 activation/upregulation has been recognized to be associated with the malignance in various types of human cancers, such as cancers in ovarium [27], gaster [28, 18], hypar [29]. The prognostic value and therapeutic potential of PAK4 has been confirmed in ovarian cancer [27]. PAK4 has been indicated to correlate with poorer survival in patients with metastatic gastric cancer [30]. Therefore, these findings suggested that PAK4 is indicative for the clinical progression and prognosis of GC. In the present study, we confirmed the upregulation of PAK4 in the tumor tissues than in the peritumor tissues in 93 cases of cervical cancers; and the PAK4 upregulation was markedly associated with the FIGO stage, lymph node metastasis, distant metastasis and histological grade of these cervical cancer patients, was significantly and independently prognostic for the overall survival time of these patients.

Accumulating evidence has confirmed the mediation of PAK4 in chemoresistance in various types of cancers. Recently, PAK4 has been indicated to enhance the survival and decrease the apoptosis of prostate cancer cells following chemotherapy [31], and to be a predictive marker of gemcitabine sensitivity in pancreatic cancer cell lines [32]. And in gastric cancer, overexpressed PAK4 has also been suggested to confer the resistance to capecitabine/cisplatin chemotherapy [30, 24]. In the present study, PAK4 overexpression posed marked amelioration of the viability reduction of cervical Hela and Caski cells post the cisplatin treatment, time-dependently, whereas the knockout of PAK4 aggravated the cisplatin-mediated viability reduction in both types of cells. Therefore, our study confirmed the mediation of PAK4 in the resistance to cisplatin in Hela and Caski cells. In addition, the PI3K/Akt-dependent pathway was implicated in the PAK4-confered cisplatin resistance in Hela and Caski cells. The phosphorylated AKT was significantly promoted by the cisplatin treatment in Hela and Caski cells. However, the promotion to the phosphorylated AKT was inhibited by the knockout of PAK4. On the other side, the PI3K/Akt inhibitor, LY294002 markedly deteriorated the cisplatin-mediated viability reduction of Hela and Caski cells. Thus, our results confirmed the involvement of PI3K/Akt-dependent pathway in the PAK4-confered cisplatin resistance.

## Conclusion

In summary, this study has confirmed the significant prognostic role of PAK4 level in cervical cancer patients and has recognized the regulatory role in cervical cancer progression. High PAK4 level is correlated with the advanced stage cervical cancer, and is an independent factor for predicting the clinical prognosis of patients with cervical cancer. Moreover, our study has indicated that PAK4 also confers the chemoresistance of cervical cancer cells in a PI3K/Akt-dependent way. Thus, our study indicates PAK4 as a promising marker for cervical cancer treatment.
